# Reversal of Ischemic Cardiomyopathy with Sca-1^+^ Stem Cells Modified with Multiple Growth Factors

**DOI:** 10.1371/journal.pone.0093645

**Published:** 2014-04-04

**Authors:** Ning Li, Zeeshan Pasha, Muhammad Ashraf

**Affiliations:** 1 Department of Physiology and Cell Biology, The Ohio State University, Columbus, Ohio, United States of America; 2 Department of Pharmacology, University of Illinois at Chicago, Chicago, Illinois, United States of America; Tokai University, Japan

## Abstract

**Background:**

We hypothesized that bone marrow derived Sca-1^+^ stem cells (BM Sca-1^+^) transduced with multiple therapeutic cytokines with diverse effects will induce faster angiomyogenic differentiation in the infarcted myocardium.

**Methods and Results:**

BM Sca-1^+^ were purified from transgenic male mice expressing GFP. Plasmids encoding for select quartet of growth factors, i.e., human IGF-1, VEGF, SDF-1α and HGF were prepared and used for genetic modification of Sca-1^+^ cells (^GF^Sca-1^+^). Scramble transfected cells (^Sc^Sca-1^+^) were used as a control. RT-PCR and western blotting showed significantly higher expression of the growth factors in ^GF^Sca-1^+^. Besides the quartet of the therapeutic growth factors, PCR based growth factor array showed upregulation of multiple angiogenic and prosurvival factors such as Ang-1, Ang-2, MMP9, Cx43, BMP2, BMP5, FGF2, and NGF in ^GF^Sca-1^+^ (*p<*0.01 *vs* ^Sc^Sca-1^+^). LDH and TUNEL assays showed enhanced survival of ^GF^Sca-1^+^ under lethal anoxia (*p<*0.01 *vs* ^Sc^Sca-1^+^). MTS assay showed significant increased cell proliferation in ^GF^Sca-1^+^ (*p<*0.05 *vs* ^Sc^Sca-1^+^). For in vivo study, female mice were grouped to receive the intramyocardial injection of 15 μl DMEM without cells (group-1) or containing 2.5×10^5^ ^Sc^Sca-1^+^ (group-2) or ^GF^Sca-1^+^ (group-3) immediately after coronary artery ligation. As indicated by *Sry* gene, a higher survival of ^GF^Sca-1^+^ in group-3 on day4 (2.3 fold higher *vs* group-2) was observed with massive mobilization of stem and progenitor cells (cKit^+^, Mdr1^+^, Cxcr4^+^ cells). Heart tissue sections immunostained for actinin and Cx43 at 4 weeks post engraftment showed extensive myofiber formation and expression of gap junctions. Immunostaining for vWF showed increased blood vessel density in both peri-infarct and infarct regions in group-3. Infarct size was attenuated and the global heart function was improved in group-3 as compared to group-2.

**Conclusions:**

Administration of BM Sca-1^+^ transduced with multiple genes is a novel approach to treat infarcted heart for its regeneration.

## Introduction

Stem cell based cell therapy offers a potentially therapeutic option for ischemic heart disease [1]. Bone marrow-derived stem cells (BMSCs) have been widely studied for use in cardiac repair due to their favorable properties including multipotency, transdifferentiation, immunomodulation and free from the risks of teratoma formation. Promising results have been reported in preclinical and clinical studies [2]–[5]. The results show that BMSCs not only differentiate into cardiomyocytes and vascular cells, but also secrete multiple growth factors and cytokines which may mediate endogenous regeneration via activation of resident cardiac stem cells and neovascularization, and reduce apoptosis [6]. Nevertheless, current evidence supports that efficacy of BMSC was limited due to the poor viability and massive death of the engrafted cells in the infarcted myocardium. The heart cell therapy with BMSC to compensate for loss of functional cardiomyocytes during the ischemic episode may be less meaningful without restoration of the regional blood flow in the ischemic myocardium. Hence, it would be practical to combine cell transplantation with therapeutic gene delivery to the heart to achieve maximum benefits of stem cell therapy.

In this study, we hypothesized that a combined approach involving BM Sca-1^+^ cells genetically modified to express multiple specific therapeutic genes including vascular endothelial growth factor (VEGF), insulin like growth factor-1 (IGF-1), hepatocyte growth factor (HGF) and stromal cell derived factor-1α (SDF-1α) would be more effective in promoting new growth and preservation of the global heart functions. The BM derived Sca-1^+^ cells would serve as reservoirs of multiple growth factors to support angiomyogenic repair of the infarcted heart. Moreover, expression of growth factors in the heart would create a gradient to favor mobilization of resident stem/progenitor cells from the BM, peripheral circulation and the heart via specific ligand/receptor interaction for participation in the angiomyogenic repair of the infarcted heart.

## Materials and Methods

### Ethics Statement

All animal experimental procedures conform to the Guide for the Care and Use of Laboratory Animals published by the US National Institutes of Health (NIH Publication #85-23, revised 1996) and were conducted according to a protocol approved by the Institutional Animal Care and Use Committee, University of Cincinnati.

### In vitro Studies

#### BM Sca-1^+^ selection

BM was harvested from 6–8 weeks old transgenic male mice expressing GFP. Sca-1^+^ cells were purified by EasyStep (Stem cell Technology Inc.) isolation kit according to the manufacturer’s instruction. Sca-1 surface marker was confirmed by flow cytometry and fluorescent immunostaining as described earlier [7] and detailed in Text S1.

#### Preparation of plasmids and nano-particle based cell transfection

Plasmids encoding for select quartet of growth factors, i.e., human IGF-1(pCMV-IGF), VEGF (pCMV-VEGF), SDF-1α (pORF-hSDF-1α) and HGF (pBLAST49-hHGF) were prepared and used for genetic modification of Sca-1^+^ cells (^GF^Sca-1^+^) as in Figure S1. The list of primers used are described in Table S1. Cells were separately transfected with one of the 4 plasmids using Polyethyleneimine (PEI, Polysciences Inc.) based on our optimized protocol as described in Text S1. After 48 hours in culture, the cells transfected with respective growth factor were pooled together and cultured for further 24 hours before use for *in vitro* as well as *in vivo* studies. Scramble transfected Sca-1^+^ cells (^Sc^Sca-1^+^) were used as control.

#### *In vitro* characterization of transfected Sca-1^+^ cells

Transfection and expression efficiencies were determined by RT-PCR, western blotting and fluorescent immunostaining 48 hours after their transfection with each plasmid.

#### Estimation of cytoprotection and cell proliferation

The cytoprotective action of growth factors transfection was assessed by treating the ^GF^Sca-1^+^ and ^Sc^Sca-1^+^ or co-cultured cardiomyocytes (CM) under anoxia with glucose and serum free DMEM. At different time points, the supernatant of different group were collected. Lactate dehydrogenase (LDH) release was measured using CytoTox-ONE™ homogeneous membrane integrity assay (Promega) as an indicator of cell membrane integrity and cell viability. The experimental protocol is detailed in Text S1.

In addition to the LDH assay, terminal dUTP Nick-End Labeling Assay (TUNEL) was performed on paraformaldehyde fixed cells with in-situ cell death detection kit (TMR red; Roche Inc.) per manufacturer’s instructions. The degree of apoptotic cell death was determined by counting total number of TUNEL positive nuclei per microscopic field (400x).

The cell proliferation was evaluated in vitro with the use of the MTS [3-(4,5-dimethylthiazol-2-yl)-5-(3-carboxymethoxyphenyl)-2-(4-sulfophenyl)-2H-tetrazolium] assay according to the manufacturer’s recommendations (Promega) as previously described [8].

#### Tube formation and cell migration assays

Conditioned medium from transfected pooled Sca-1^+^ cells were used for matrigel angiogenesis assay and transwell cell migration assay as detailed in Text S1.

### *In vivo* Studies

#### Experimental animal model of myocardial infarction

Myocardial infarction model was developed in 8–12 weeks old female C57BL/6J mice as described earlier [9]. Briefly, the animals were anesthetized (Ketamine/Xylazine 0.05 ml intraperitoneally), intubated, and mechanically ventilated. Minimally invasive thoracotomy was performed for permanent ligation of left anterior descending coronary artery. Myocardial ischemia was confirmed by color change of left ventricular wall. The animals were grouped to receive 15 μl DMEM without cells (group-1) or containing 2.5×10^5^ ^Sc^Sca-1^+^ cells (group-2) or growth factor transfected pooled cells (group-3) by direct intramyocardial injections in and around the infarction area. The chests were closed and the animals were allowed to recover. Buprenex (0.1 mg/kg per 12 hours) was administered for 24 hours to alleviate pain. The animals were euthanized on 4 days and 4 weeks after their respective treatment. The heart tissue samples were used for molecular, histological and immunohistological studies.

#### Estimation of cells survival post transplantation

PCR for *sry*-gene expression [7] was performed on myocardial tissue samples from various treatment groups of the animals 4 days after their respective treatment (n = 4 per group).

#### Histological studies

Histological and immunohistochemical studies were carried out as detailed in Text S1. The antibodies and their concentrations used are given in Table S2.

#### The heart function studies and infarction size measurement

The heart function evaluation was performed in all the animals by transthoracic echocardiography 4 weeks after their respective treatment (n = 7/group) as described in Text S1. Histological tissue sections were stained with H&E and Masson’s Trichrome for infarction size measurement.

### Statistical Analysis

All the data were described as mean±SEM. To analyze the data statistically, we performed Student’s *t*-test and one-way ANOVA with post-hoc analysis and considered a value of *p*<0.05 as statistically significant.

## Results

### In vitro Studies

#### Efficiency of growth factor transfection in purified BMSca-1^+^

**Figure 1:**
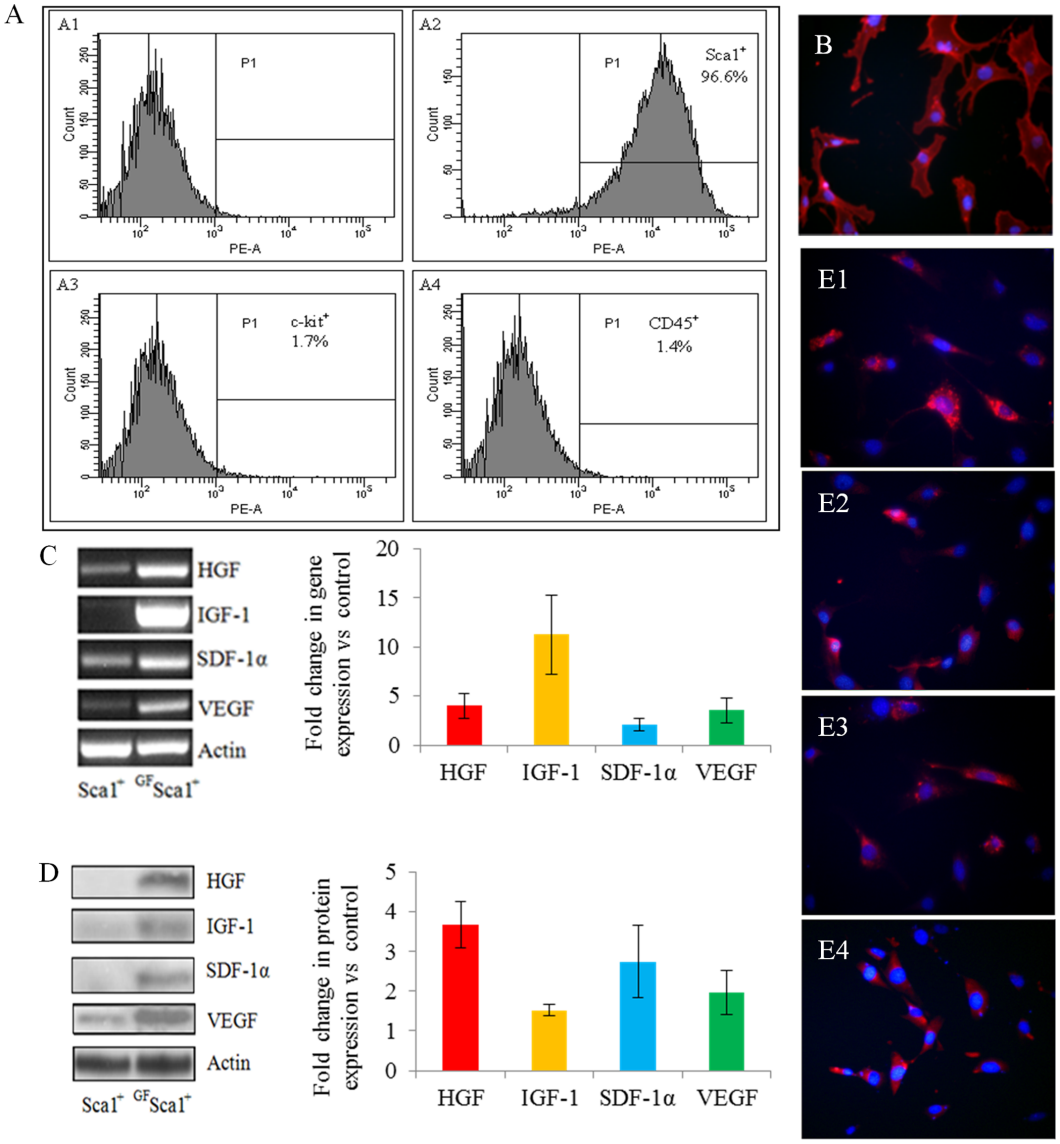
Flow cytometry of the purified mouse BM Sca-1^+^ cells for surface markers analysis. (A) unlabeled Sca-1^+^ cells (control; A1), labeled cells showing 96.6% Sca-1^+^ (A2),1.7% c-kit^+^(A3), and 1.4% CD45^+^(A4) cell populations. (B) Immunostaining of cells for Sca-1 antigen (red = Sca-1; blue = DAPI; magnification = 400x). (C–E) Representative figures of *in vitro* characterization of BM Sca-1^+^ cells transfected for expression of the respective transgene. RT-PCR (C) and western blot (D) for HGF, IGF-1, SDF-1α, and VEGF transgene expression *in vitro*. In each case, non-transfected native Sca-1^+^ cells were used as a control. Densitometry showed significantly up-regulated expression of the respective growth factor gene and protein in the transfected Sca-1^+^ cells 48 hours after transfection. (E) Fluorescence images of the Sca-1^+^ cells immunostained for respective growth factor HGF (E1, red), IGF-1(E2, red), SDF-1α (E3, red), VEGF (E4, red). DAPI was used to visualize the nuclei (blue, magnification = 400x).

BM Sca-1^+^ were successfully isolated from male donor mice. Flow cytometry (Figure-1 Panel A; A1–A4) and fluorescent immunostaining (Figure-1B) demonstrated more than 96% pure Sca-1^+^ population. Plasmid encoding for human IGF-1(pCMV-IGF), VEGF (pCMV-VEGF), SDF-1α (pORF-hSDF-1α) and HGF (pBLAST49-hHGF) were successfully constructed and used for transfection of Sca-1^+^. Expression of the respective transgene was increased in Sca-1^+^ after transfection with the individual growth factor plasmid which was evident from RT-PCR (Figure 1C), western blotting (Figure 1D) and fluorescence immunostaining (Figure 1E, E1–E4).

#### Gene expression by ^GF^Sca-1^+^

**Figure 2:**
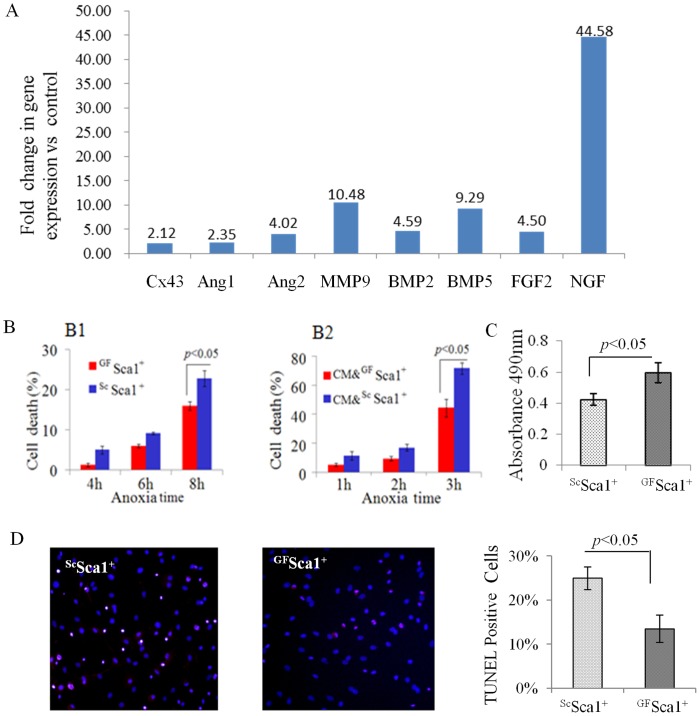
(A) Real-time PCR based growth factor array showing up-regulation of multiple angiogenic and pro-survival factors including connexin-43 (Cx43), angiopoietin-1 & 2 (Ang-1 & Ang-2), matrix metalloproteinase-9 (MMP9), Bone morphogenetic protein 2 & 5 (BMP2 & 5), fibroblast growth factor-2 (FGF-2), and Nerve growth factor (NGF) in pooled ^GF^Sca-1^+^ cells compared to control Sca-1^+^ cells. (B; B1–B2) LDH release assay showed significant reduction of cell death in ^GF^Sca-1^+^ as compared to ^Sc^Sca-1^+^ after 8 hours cultured under lethal anoxia (B1) and co-cultured cardiomyocytes after 3 hours under lethal anoxia (B2). (C) MTS assay was used to determine proliferative activity in vitro. The proliferation rate of ^GF^Sca-1^+^ group was significantly higher as compared to ^Sc^Sca-1^+^ (0.59±0.06 vs. 0.42±0.04 absorbance at 490 nm) (D) TUNEL staining confirmed that 8 hours lethal anoxia caused higher TUNEL positivity in ^Sc^Sca-1^+^ as compared to ^GF^Sca-1^+^ cells.

Besides multi-fold increase by overexpression of the four growth factors, real-time PCR using growth factor array showed up-regulation of multiple angiogenic and pro-survival factors including angiopoietin-1 & 2 (Ang-1 & Ang-2), matrix metalloproteinase-9 (MMP9), connexin-43 (Cx43), bone morphogenetic protein-2 & -5 (BMP-2 & 5), fibroblast growth factor-2 (FGF-2), and nerve growth factor (NGF) in pooled ^GF^Sca-1^+^ cells compared to native Sca-1^+^ cells (Figure 2A).

#### Effect of ^GF^Sca-1+ on cytoprotection, proliferation and angiogenesis

Transfection of Sca-1^+^ with growth factors was protective against anoxia under co-culture conditions and prevented cell death. LDH release, a marker of cellular injury was significantly reduced in ^GF^ Sca-1^+^ and co-cultured cardiomyocytes (CM) (Figure 2B). Also TUNEL positive cells were reduced in ^GF^Sca-1^+^ (13.52% ±3.08%) as compared to ^Sc^Sca-1^+^ cells (24.98% ±2.60%, *p*<0.05, Figure 2D) under 8 hours lethal anoxia. To examine whether growth factor transfection had any effect on proliferation rate, MTS assay was used to determine proliferative activity *in vitro*. The proliferation rate of ^GF^ Sca-1^+^ group was significantly higher compared with ^Sc^Sca-1^+^ (0.59±0.06 *vs*. 0.42±0.04 absorbance at 490 nm, Figure 2C).

**Figure 3:**
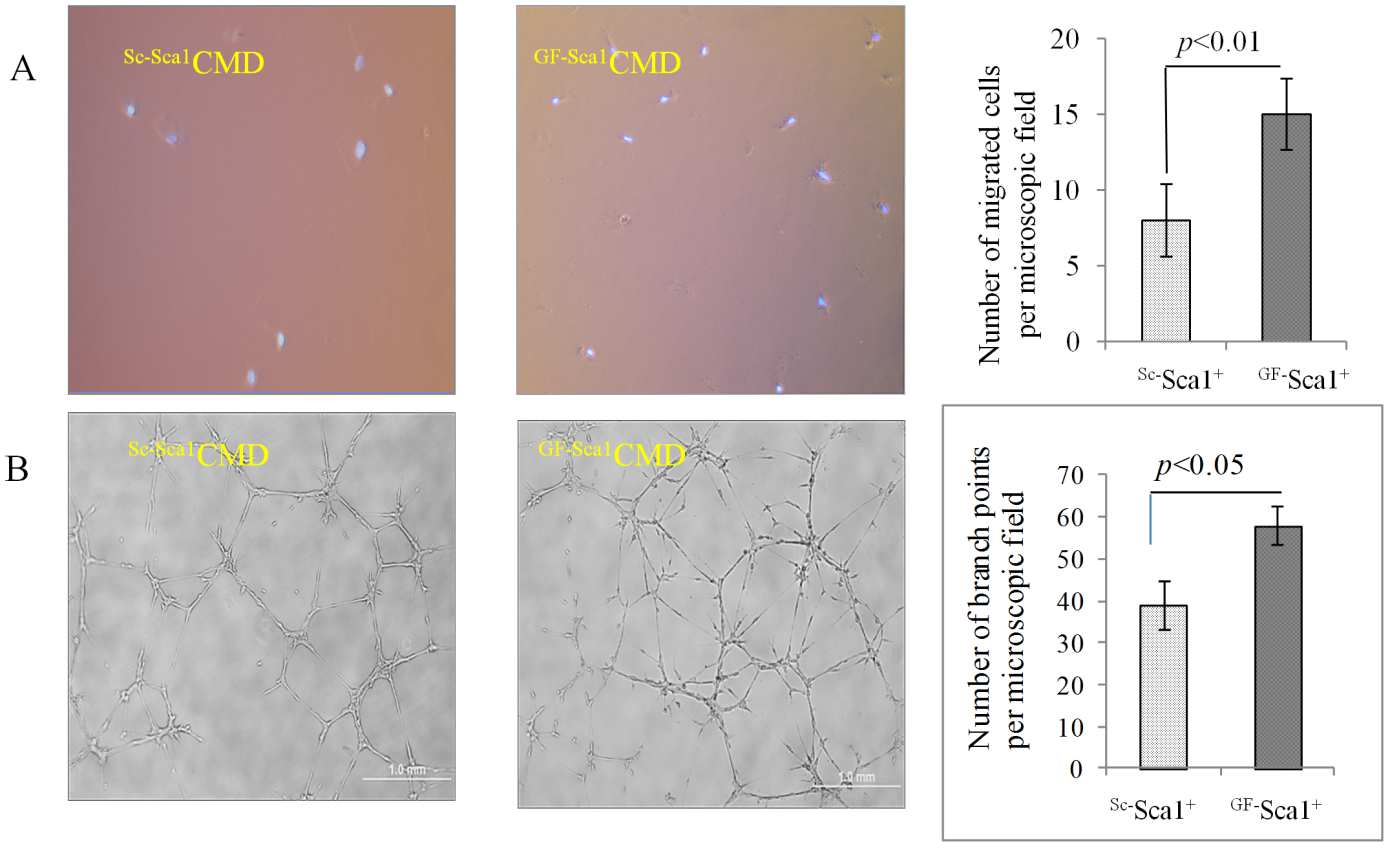
Cell migration and angiogenic effects of CMD *in vitro*. (A)Trans-well cell migration assay using HUVECs showed that the number of migrating cells per microscopic field (200x) in response to ^GF-Sca1^CMD was significantly higher compared to ^Sc-Sca1^CMD as a control (15±2.38 *vs* 8±2.35, *p*<0.01). Nuclei were stained with DAPI (blue). (B) Tube formation assay on matrigel using HUVECs treated with conditioned medium (CMD) from ^GF^Sca-1^+^ cells (^GF-Sca1^CMD) or ^Sc^Sca-1^+^ cells (^Sc-Sca1^CMD). ^GF-Sca1^CMD showed significantly higher number of branch points per microscopic field (100x) as compared to ^Sc-Sca1^CMD.

The conditioned medium from ^GF^ Sca-1^+^ (^GF-Sca1^CMD) also promoted human umbilical vein endothelial cells (HUVEC) migration *in vitro* in the trans-well migration system as compared with medium from ^SC^Sca-1^+^(^Sc-Sca1^CMD) (15±2.38 *vs* 8±2.35, *p*<0.01, Figure 3A). Similarly, *in vitro* tube formation assay on matrigel showed higher branch points (58±5.8) per microscope field (100x) at 16 hours after incubation with ^GF-Sca1^CMD as compared with ^Sc-Sca1^CMD (39±4.6, *p*<0.05, Figure 3B).

### In vivo Studies

#### Sca-1^+^ transplantation and differentiation in the heart

**Figure 4:**
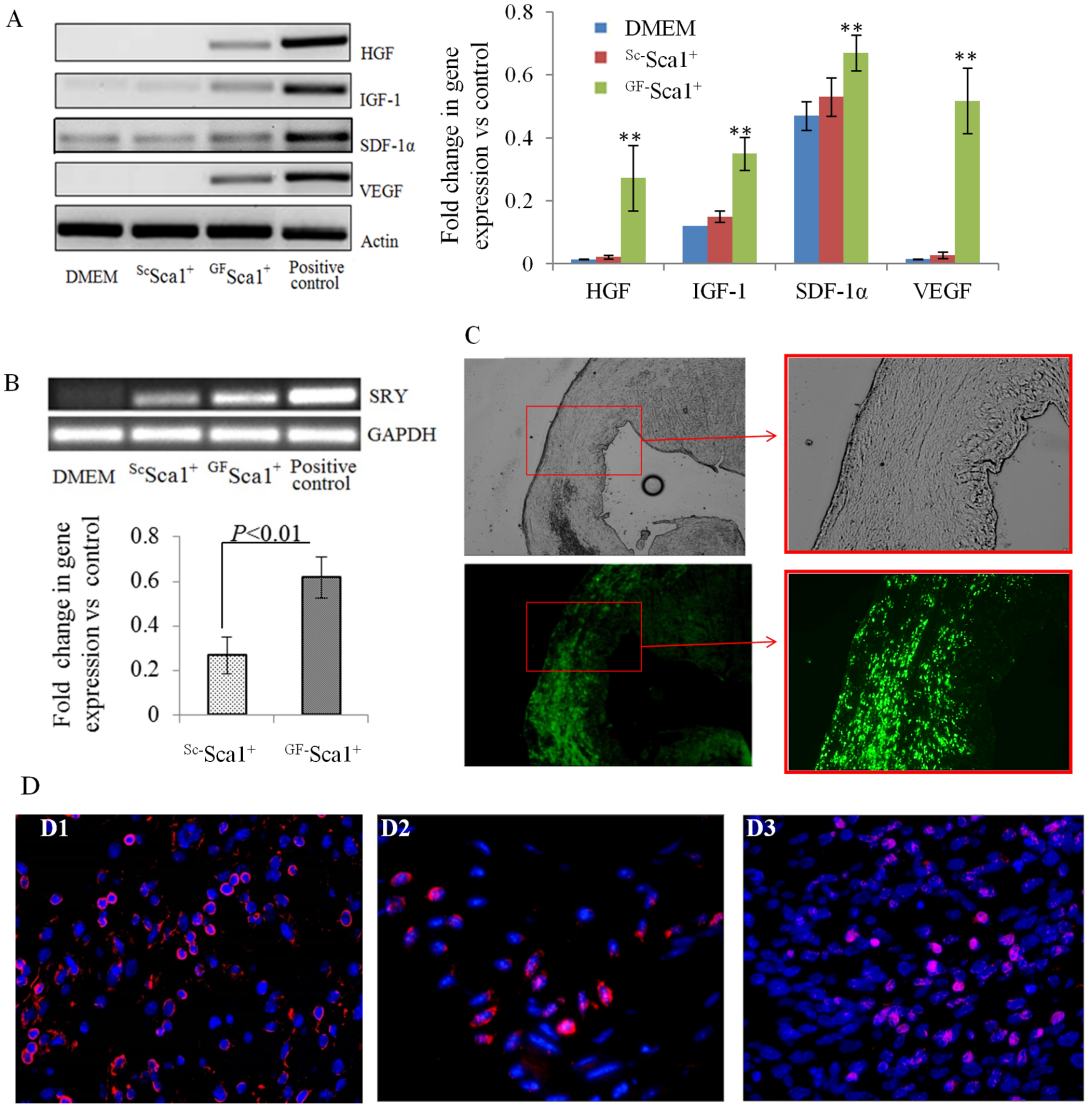
Cell survival and release of growth factors at 4 day post transplantation. (A) RT-PCR using the heart tissue samples on day 4 from different treatment groups. The results showed continued expression of each growth factor until day 4 post transplantation. (**P<0.01, ^GF^Sca-1^+^ group *vs* control groups). (B) *Sry*-gene expression in female mouse heart revealed extensive survival of the male donor cells in ^GF^Sca-1^+^ group as compared to ^Sc^Sca-1^+^ group. (C) Fluorescent immunostaining for GFP expression showed extensive presence of ^GF^Sca-1^+^ cells in the infarct and peri-infarct regions at 4 days after transplantation (magnification 40x, 100x). (D) Immunostaining for c-kit (D1, red), CXCR4 (D2, red) and MDR1(D3, red) showed extensive presence of mobilized cells in the infarct and peri-infarct regions at 4 days after ^GF^Sca-1^+^ transplantation (magnification 200x). The nuclei are stained with DAPI (blue).

RT-PCR of heart tissue samples showed higher expression of transfected growth factors including HGF, IGF-1, SDF-1α and VEGF 4 days after engraftment (Figure 4A). The transplanted male ^GF^Sca-1^+^ showed better survival in the infarcted heart. PCR for *sry*-gene in female mice heart on day 4 revealed 2.3 times increased survival of the donor male cells in ^GF^ Sca-1^+^ group as compared to ^Sc^Sca-1^+^ group (*P*<0.01, Figure 4B). We did not observe *sry*-gene expression in DMEM injected female animal hearts which served as a negative control. These results were supported by immunohistological studies which confirmed extensive presence of GFP^+^ positive cells at the site of cell graft in ^GF^ Sca-1^+^ group at day 4 after transplantation (Figure 4C).

#### Stem/progenitor cells homing into myocardium

The histological sections of heart tissues from different treatment groups were immunostained for the presence of stem and progenitor cells of various lineages which were mobilized into the infarcted heart. Figure 4D shows numerous c-Kit^+^, CXCR4^+^, MDR1^+^ (Figure 4D; D1–D3) cells respectively which mobilized into the infarcted heart in ^GF^ Sca-1^+^ treatment group. The number of mobilized ckit^+^ cells was significantly higher in group-3 as compared to group-2 (55.50±9.87 *vs.* 20.88±8.53/microscope field, *p*<0.05).

#### Histological evaluation of transplanted cells in the infarcted heart

**Figure 5:**
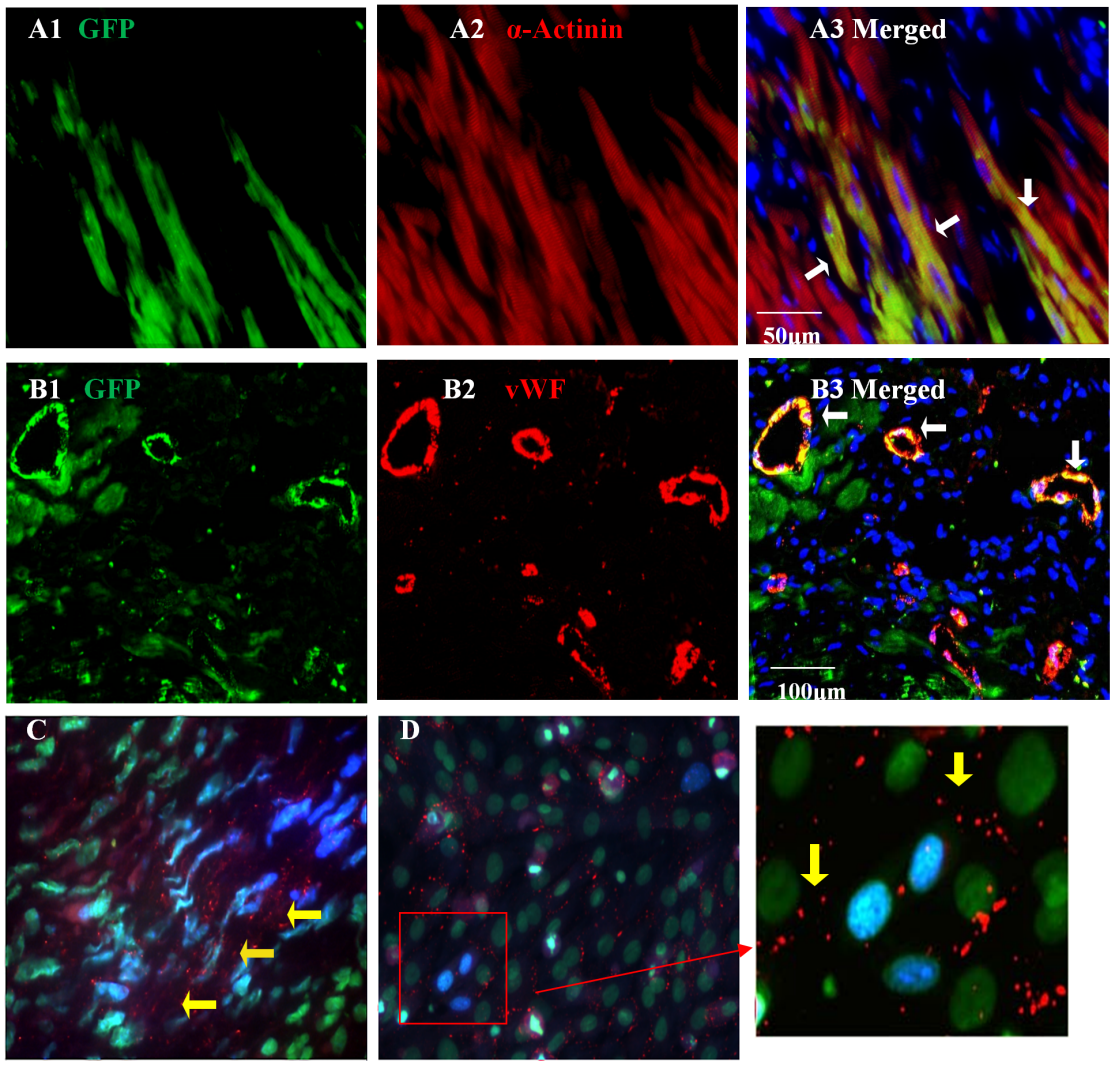
Microscopic images from recipient mouse hearts 4 weeks post-transplantation. Fluorescence immunostaining of the heart tissue revealed increased angiomyogenic differentiation of the transplanted Sca-1^+^ in the infarct and peri-infarct regions 4 weeks after transplantation (GFP (green 5A1,5B1), α-actinin (red; Figure 5A2) and vWF (red; Figure 5B2)). Arrows show the merged color of red and green indicating the colocalization of α-actinin and GFP (5A3) or vWF and GFP (5B3). The nuclei were stained with DAPI (blue) (C) Immunostaining of the heart tissue revealed Cx43 (red) expression between^ GF^Sca-1^+^ and the host cardiomyocytes (indicated by yellow arrows). (D) Cx43 (red) expression between ^GF^Sca-1^+^ and the co-cultured cardiomyocytes at day 4 after co-culture. (C–D) ^GF^Sca-1^+^ stem cells were prelabeled with DAPI (blue), and the nuclei were double stained with SYTOX (green).

Immunostaining of the heart tissue for subcellular structures revealed increased angiomyogenic differentiation of the transplanted Sca-1^+^ in the infarct and peri-infarct regions 4 weeks after transplantation (GFP, green; Figure 5A1, B1), α-actinin (red; Figure 5A2) and vWF (red; Figure 5B2). Fluorescence immunostaining also showed Cx43 positivity for gap junctions formed between^ GF^Sca-1^+^ and the host cardiomyocytes in the infarcted area (Figure 5C). These observations were consistent with our *in vitro* data that ^GF^Sca-1^+^ showed two fold increase in Cx43 (Figure 2A) and Cx43 formation between co-cultured ^GF^ Sca-1^+^ and cardiomyocytes (Figure 5D).

#### ^GF^Sca-1^+^ improve angiogenesis in the infarcted myocardium

**Figure 6:**
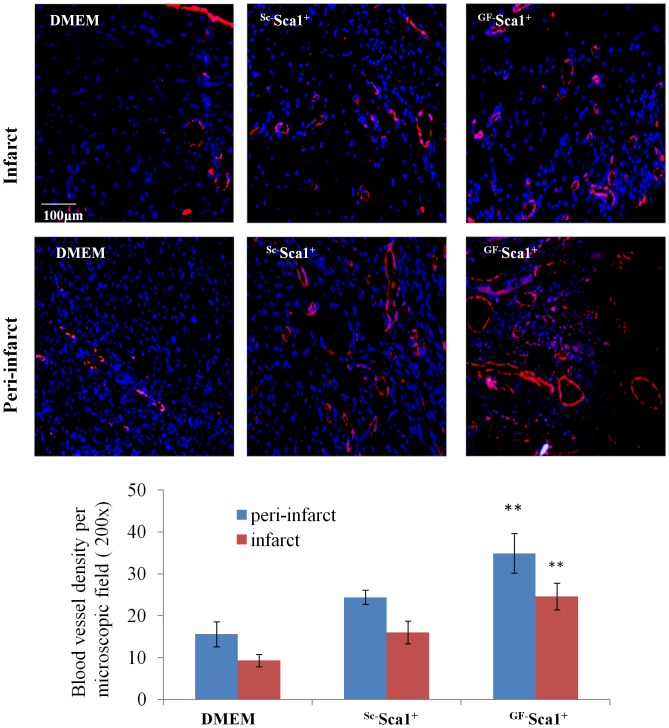
Blood vessel density analyzed by fluorescence immunostaining for vWFactor-VIII (red). Blood vessel density was significantly higher in ^GF^Sca-1^+^ group both in the infarct and peri-infarct regions as compared to control groups (**p<0.01, magnification 200x).

Blood vessel density was measured in the infarct and peri-infarct regions after immunostainning of histological sections with vWF (red) to detect vascular structures. A large number of blood vessels were observed in the cell transplanted areas in both ^GF^ Sca-1^+^ and ^Sc^Sca-1^+^ treatment groups. Blood vessel count per microscope field (200x; Figure 6) was the highest in both peri-infarct (34.9±4.7) and infarct (24.6±3.2) regions in ^GF^ Sca-1^+^ group (*P*<0.01) as compared to the peri-infarct and infarct regions in ^Sc^Sca-1^+^ group (24.4±1.7 and 16±2.7 respectively) and in DMEM group (15.6±2.9 and 9.3±1.5 respectively, Figure 6).

#### Infarct size and heart function

**Figure 7:**
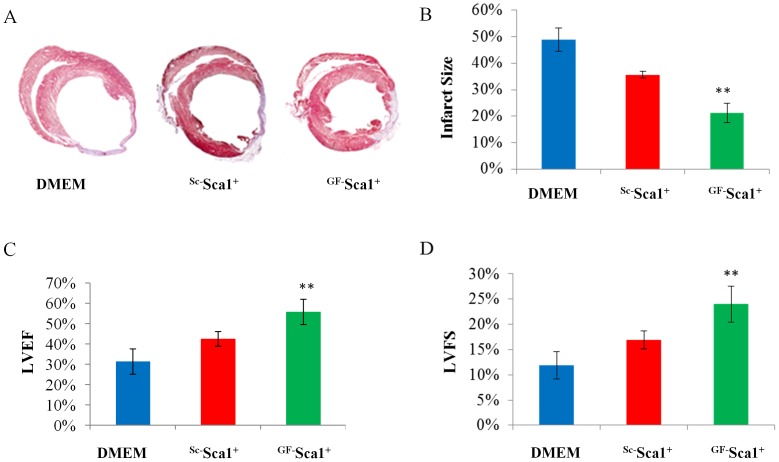
Masson trichrome staining was performed to evaluate the infarction size. Significant attenuation of infarct size was observed in ^GF^Sca-1^+^ group as compared to other groups. Indices of the left ventricular contractile function including LVEF and LVFS were significantly preserved in ^GF^Sca-1^+^ group at 4 weeks after cell engraftment as compared to other groups(**P<0.01).

Histological sections at mid-papillary muscle level followed by Masson’s trichrome staining showed transmural infarction in all the animals at 4 weeks after coronay artery ligation (Figure 7A). Noticeable left ventricle wall thinning was observed at 4 weeks in DMEM treated group whereas left ventricle wall thickness was considerably retained in ^GF^Sca-1^+^ group. Infarct size was significantly lower at 4 weeks in ^GF^Sca-1^+^ group (21.2% ±3.6%; *p*<0.01) as compared to DMEM group (48.9%±4.3%) and ^Sc^Sca-1^+^ group (35.6% ±1.3%, Figure 7B). Transthoracic echocardiography of animals at 4 weeks after their respective treatment showed significant differences in the indices of left ventricle function amongst various treatment groups. Left ventricle ejection fraction (55.9±6.2%) and left ventricle fractional shortening (24.0±3.6%) were significantly improved in ^GF^Sca-1^+^ group as compared to ^Sc^Sca-1^+^ group (42.6±3.6% and 16.9±1.7% respectively) and DMEM injected group (31.4±6.2% and 11.9±2.7% respectively, *p*<0.01, Figure 7C–D).

## Discussion

For last several years, BMSCs have been extensively used to regenerate infarcted myocardium [10], [11] with heterogeneous results. More recently attempts have been made to render these cells smarter by modifying them exogenous growth factors or gene transfer strategies. Previously we have shown the effectiveness of several individual growth factors for treatment of experimental myocardial infarction [12]–[15] and in this study we transfected the BM Sca-1^+^ cells with individual transgenic plasmids and then pooled the transfected cells before transplantation. This multimodal non-viral gene therapy approach resulted in activation and participation of the intrinsic ligand/receptor systems: VEGF/VEGFR, HGF/cMet, IGF-1/IGF-1R and SDF/CXCR4, in the repair processes of infarcted myocardium. Our multipronged strategy activated the multiple signaling mechanisms resulting in reduced cell apoptosis, increased cell proliferation, massive stem/progenitor cell homing to the site of ischemic injury. The transplanted and mobilized cells appeared to differentiate into cardiomyocytes in the infarcted area with significant improvement in the left ventricular ejection fraction.

The rationale of selecting various growth factors was based on their distinct functional role in the myocardial biology. These growth factors have pleiotropic functions. IGF-1 interacts with its IGF-1R receptors to impart its cytoprotective, pro-proliferative and pro-differentiation effects [16], [17]. We have reported that IGF-1 preconditioning of Sca-1^+^ cells promotes Cx43 induction and their translocation into mitochondria for its dual role of stem cell integration as well as protection post-engraftment [7]. HGF is a mesenchyme derived pleiotropic factor that is thought to mediate the interaction between epithelial and endothelial cells via autocrine and paracrine functions, promoting angiogenesis. In addition, HGF exerts its anti-cell death effects by blocking apoptosis [18]. The HGF receptor is a membrane spanning tyrosine kinase encoded by the c-Met proto-oncogene [19]. Myocardial HGF/c-Met is upregulated in the heart following myocardial infarction [20], [21]. HGF does not influence the mitosis of cardiac cells, but prevents post-infarction remodeling of heart. Administration of IGF/HGF improved cardiomyocyte survival, and reduced fibrosis and cardiomyocytes reactive hypertrophy. It significantly activated resident endogenous cardiac stem/progenitor cells and fostered the generation of new myocytes and microvasculature in infarcted and peri-infarct/border areas [22]. VEGF is well known for its ability to stimulate proliferation of endothelial cells in vitro and neovasculogenesis in the ischemic heart in vivo [23], [24]. In addition VEGF, an angiogenic growth factor can also promote differentiation of stem cells into cardiomyocytes and endothelial cells [25], [26]. SDF-1α is a ligand for CXCR4 receptors expressed on BMSC. SDF was transiently upregulated in infarct and peri-infarct regions in experimental animal models of myocardial infarction and SDF-1/CXCR4 interactions played a crucial role in the recruitment of BMSCs to the infarcted myocardium [8]. The SDF-1/CXCR4 axis seems to be a novel therapeutic approach to improve post-infarction therapy by attracting circulating stem cells to the site of injury where they differentiated into cardiac cells in the infarcted heart [27]. We have previously shown that transplantation of MSCs overexpressing IGF-1 caused massive stem cell mobilization into infarcted hearts through paracrine signaling of SDF-1α resulting in extensive angiogenesis with better cardiac function [12]. A recent study has suggested that SDF-1α and VEGF form a synergistic angiogenic pathway that is critical for endothelial progenitor cell induced neovascularization [28].

Given the distinct functional role of these cytokines, our novel strategy of multiple growth factor transgene delivery is aimed to harness their combined beneficial effects for treatment of infarcted heart. This study provides evidence that the selected quartet of growth factors created a growth factor gradient for mobilization of recipient’s own pool of BMSC, EPC and resident cardiac stem cells for participation in the repair process. In addition MSCs can secrete large amounts of paracrine factors which are angiogenic and anti-apoptotic, alter the restoration of extracellular matrix and also recruit endogenous stem cells [29], [30], [31]. In this study, we also observed significant upregulation of matrix metalloproteinases (MMP), bone morphogenetic protein (BMP), nerve growth factor (NGF), and fibroblast growth factor (FGF).

MMPs are important for cell migration, invasion, proliferation, and apoptosis prevention. They regulate many developmental processes, including branching morphogenesis, angiogenesis, wound healing, and extracellular matrix degradation [32]. MMP-9 induced in BM cells, releases soluble kit-ligand (sKitL), permitting the transfer of stem cells from the quiescent state to proliferative niche favoring differentiation and reconstitution of the stem/progenitor cell pool. SDF-1 and VEGF both can up-regulate MMP-9 expression, and cause shedding of sKitL and recruitment of c-Kit^+^ stem/progenitors [33]. BM-derived MMP-9 plays an important role in BM cell mobilization and focal angiogenesis in response to VEGF stimulation [34]. BMPs are part of the transforming growth factor β (TGF-b) superfamily [35] and comprise of a large, evolutionarily conserved family of secreted signaling molecules that are required for numerous developmental processes. BMP-2 is known to play an important role in the process of heart development [36]. BMP signaling also cooperates with VEGF signaling to affect the process of angiogenesis [37]. In vitro, BMP-2 stimulates proliferation of human aortic endothelial cells (HAEC) [38] and pulmonary aortic endothelial cells (PAEC) [39]. BMP-2 also increases the migration and tube formation of human microvascular endothelial cells (HMEC) [40] and human umbilical vein endothelial cells (HUVEC) [38]. NGF is a secreted glycoprotein of the neurotrophin family. It promotes angiogenesis and cardiomyocyte survival, which are both desirable for postinfarction myocardial healing. Recent findings demonstrate that the co-transfection of hNGF+VEGF genes in BMSCs can enhance the angiogenic effect *in vivo* [41]. NGF elicits its biological effects mainly by binding the high affinity TrkA receptor (tropomyosin-related receptor A). The prosurvival/proangiogenic Akt/Foxo pathway mediated the therapeutic benefits of NGF. NGF gene transfer ameliorated endothelial cells and cardiomyocyte survival, promoted neovascularization and improved myocardial blood flow and cardiac function in the infarcted myocardium [42]. FGFs are members of a family of polypeptides and FGF receptor (FGFR) are a family of transmembrane tyrosine kinase. FGF and FGFR system plays a significant role in mitogenic and angiogenic activity. FGF-2 can be induced by VEGF while FGF2 in turn can also induce VEGF expression. Both VEGF and FGF2 activate the Erk-1/2 pathway and share some similarities [43]. Thus FGF-2 is an important regulator of cell proliferation, angiogenesis, collagen synthesis, myocyte hypertrophy, scar contraction, and ultimately improvement of left ventricular contractile function [44]. FGF-2 also plays a pivotal role in the self-regeneration of the heart by accelerating the mobilization and differentiation of resident stem cells for cardiac repair [45]. More importantly, we observed higher Cx43 expression in ^GF^Sca-1^+^ transplanted animals by real time PCR and immunostaining. This is corroborated with in vitro data showing extensive Cx43 formation at the cell-cell junctions of ^GF^Sca-1^+^ co-cultured with neonatal cardiomyocytes. There are few reports on Cx43 expression by stem cells post-engraftment and their electromechanical coupling with host cardiomyocytes. However, in the present study, ^GF^Sca-1^+^ expressed significant amounts of Cx43 until 4 weeks post engraftment, which most likely improved the cells survival and differentiation.

Despite promising results, there are several limitations to this study. We used human growth factors (GFs) for pre-treatment of the BMSCs isolated from mice, although structural homology of these GFs is very high between humans and mice. Sca-1 is a cosignaling molecule that can modify the signaling capacity of receptor complex [46]. To evaluate a single factor in comparison with multiple growth factor requires thorough analysis. Because we treated BMSCs with a combination of four GFs and used non-viral method, it may limit the efficiency of individual GF effect and the understanding of underlying mechanism of individual GF. We did not look into the effect of single growth factor transduction on the gene expression of other growth factors. Although a significant increase in blood vessel density and decrease in infarction area was observed however, the role and fate of the mobilized cells could not be determined due to lack of specific markers on the cells. The cells which co-expressed GFP and cardiac muscle marker were not numerous and these cells may be the consequence of transdifferentiation of BMSCs into cardiomyocyte-like cells, or the result of cell fusion of BMSCs with host cardiomyocytes, or both [47], [48]. It is less convincing that low frequent transdifferentiation or cell fusion may contribute to significant cardiac function improvement in this study. The cytokines/growth factors exhibit a wide range of other functions (eg, stem/progenitor cell mobilization, recruitment, antiapoptotic activity, paracrine signaling and proangiogenic effects [49] thus, it is unclear to what extent the induction of cardiac differentiation by stem cells contributed to the therapeutic benefits. In a recent study on cardiac stem cells [50], a detailed mechanistic insight for the understanding of myocardial homeostasis and tissue repair has been discussed.

The present data provide supportive evidence that the gene delivery and paracrine effects mediated by ^GF^Sca-1^+^ are significant in attenuation of ventricular remodeling and the improvement of cardiac function during cell transplantation. Given the fact that tissue damage and regeneration are complex in nature, it is more likely that multiple growth factors and trophic factors acting in synergy are involved in tissue healing. Simultaneous use of BMSCs transfected with multiple cytokines with diverse effects is a novel approach for cardioprotection and regeneration of the infarcted myocardium.

## Supporting Information

Figure S1 **Plasmid sequence of human SDF-1α, HGF, IGF-1, and VEGF used for transgenic overexpression of the respective growth factor ligand in Sca-1^+^.**
(DOC)

Table S1 **Primers for conventional RT-PCR.**
(DOC)

Table S2 **Primary antibodies used for western blotting and immunohistochemistry.**
(DOC)

Text S1 **Supporting methods.**
(DOC)
