# Correction: Reversal of Ischemic Cardiomyopathy with Sca-1^+^ Stem Cells Modified with Multiple Growth Factors

**DOI:** 10.1371/journal.pone.0229293

**Published:** 2020-02-11

**Authors:** Ning Li, Zeeshan Pasha, Muhammad Ashraf

The RT-PCR images in Fig 4A of this article [1] were prepared by splicing together non-adjacent lanes of the same original gel images. The original gel images are provided here in S1 File and the RT-PCR quantification data underlying the graph in Fig 4A are provided in S2 File. In S1 File, the RT-PCR image data included in the figure panels are outlined in yellow.

The authors note that the original laboratory records for this project are held by the University of Cincinnati, where this study was completed. The data underlying results reported in this article are available upon request from the corresponding author.

As of the date of this notice, the corresponding author (MA) is affiliated with the Department of Medicine, Medical College of Georgia, Augusta, Georgia, USA.

## Supporting information

S1 File Original RT-PCR gel images used to prepare Fig 4A. (XLSX)

S2 File Raw quantitative data underlying the graph in Fig 4A. (XLSX)
