# Supplementary material for: Reversal of Ischemic Cardiomyopathy with Sca-1+ Stem Cells Modified with Multiple Growth Factors
Source: PLoS One. 2014 Apr 4;9(4):e93645. doi: 10.1371/journal.pone.0093645 (PMC3976296; doi:10.1371/journal.pone.0093645)

**Figure S1.** Plasmid **s**equence of human SDF-1α, HGF, IGF-1, and VEGF used for transgenic overexpression of the respective growth factor ligand in Sca-1^+^.


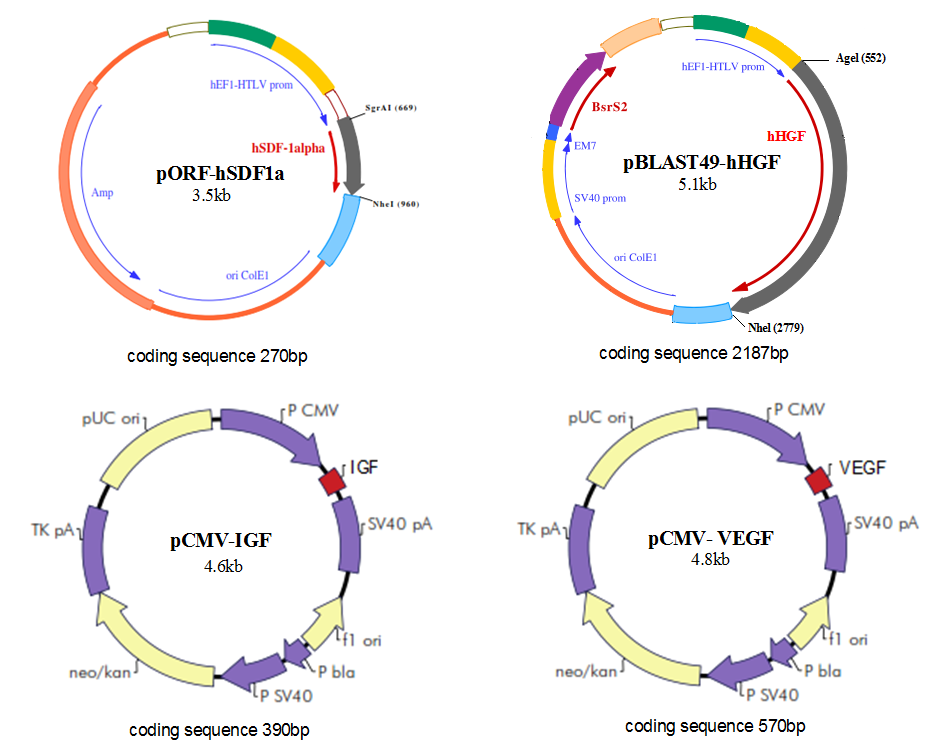

Supplement: Figure S1 — Plasmid sequence of human SDF-1α, HGF, IGF-1, and VEGF used for transgenic overexpression of the respective growth factor ligand in Sca-1+. (DOC) [file pone.0093645.s001.doc]
