# Supplementary material for: Reversal of Ischemic Cardiomyopathy with Sca-1+ Stem Cells Modified with Multiple Growth Factors
Source: PLoS One. 2014 Apr 4;9(4):e93645. doi: 10.1371/journal.pone.0093645 (PMC3976296; doi:10.1371/journal.pone.0093645)
Supplement: Table S1 — Primers for conventional RT-PCR. (DOC) [file pone.0093645.s002.doc]

**Table SI: Primers for conventional RT-PCR**

a) IGF-1 (150 bp): forward, 5' TCTGAGGAGGCTGGAGATGT 3'

reverse, 5' GTTCCGATGTTTTGCAGGTT 3'

b) VEGF (226 bp): forward, 5' CAATGATGAAGCCCTGGAGT 3'

reverse, 5' TTTCTTGCGCTTTCGTTTTT 3'

c) hSDF-1α (161 bp): forward, 5′CATGAACGCCAAGGTCGTG' 3

reverse, 5′ TCCAGGTACTCCTGAATCC 3’

d) HGF (207 bp): forward, 5’ TATTTACGGCTGGGGCTACA 3’

reverse, 5’ ACGACCAGGAACAATGACAC 3’
