# Supplementary material for: Reversal of Ischemic Cardiomyopathy with Sca-1+ Stem Cells Modified with Multiple Growth Factors
Source: PLoS One. 2014 Apr 4;9(4):e93645. doi: 10.1371/journal.pone.0093645 (PMC3976296; doi:10.1371/journal.pone.0093645)
Supplement: Table S2 — Primary antibodies used for western blotting and immunohistochemistry. (DOC) [file pone.0093645.s003.doc]

**Table S2.**

Primary antibodies used for Western blotting and immunohistochemistry.

Antibody Dilution Source

actin 1:2000 Santa Cruz Biotech

c-kit 1:100 Chemicon

Connexin-43 1:50 Santa Cruz Biotech

CXCR4 1:50 Calbiochem

HGF 1:200 R&D Systems

IGF-1 1:500 AbCam

MDR1 1:50 AbCam

SDF-1α 1:500 Cell Signaling Tech

Smooth muscle actinin 1:50 Sigma

vWillebrand Factor-VIII 1:100 Dako

VEGF 1:200 Santa Cruz
