# Supplementary material for: Reversal of Ischemic Cardiomyopathy with Sca-1+ Stem Cells Modified with Multiple Growth Factors
Source: PLoS One. 2014 Apr 4;9(4):e93645. doi: 10.1371/journal.pone.0093645 (PMC3976296; doi:10.1371/journal.pone.0093645)
Supplement: Text S1 — Supporting methods. (DOC) [file pone.0093645.s004.doc]

**Supporting information**

**Supporting methods**

**Selection of bone marrow derived Sca-1^+^**

Bone marrow was harvested from young (6-8 weeks old) transgenic male mice expressing GFP by flushing the cavities of femurs and tibias with DMEM. Bone marrow from each mouse was plated into one 100 mm culture dish and cultured in DMEM plus 20% fetal bovine serum (FBS). Fresh medium was added every 3 days. Adherent cell cultures at 70% confluence were harvested and selected for Sca-1^+^ cell population using EasySep mouse Sca-1 positive selection kit (Stem cell Technologies Inc., Vancouver, Canada) as per manufacturer’s instructions. The selected Sca-1^+^ cells were maintained and expanded for no more than 5 passages for later studies.

**Flowcytometry.**

The cells were harvested with Accutase solution (EMD Millipore Corporation, Billerica, MA, USA). 1x10^6^ cells were then re-suspended with 100μl FACS solution (PBS containing 1% bovine serum albumin (BSA) and 1mM EDTA). After blocking for nonspecific binding, the cells were incubated with 0.5μg PE-conjugated anti-Sca-1 (Cedarlane Laboratories Ltd, Hornby, Canada) at 4°C for 15-min. After washing twice with FACS solution, cells were resuspended in 500μl FACS solution and analyzed by flow cytometry (FACSCalibur, BD Bioscience, San Jose, CA,USA). Unlabelled cells were used as reference and whole bone marrow stem cells or unrelated cells were incubated with anti Sca-1 to confirm the specificity of the labeling.

**[Preparation of IGF-1, VEGF, hSDF-1α, HGF plasmid](http://www.sciencedirect.com/science?_ob=ArticleURL&_udi=B6WK6-4N0X604-1&_user=2629161&_coverDate=04%2F30%2F2007&_rdoc=1&_fmt=full&_orig=search&_cdi=6898&_sort=d&_docanchor=&view=c&_acct=C000058264&_version=1&_urlVersion=0&_userid=2629161&md5=ac301e73d4ec8d7377318645e93fb175#secx4)s**

We used an expression vector containing hSDF-1α gene (pORF-hSDF-1α) (InvivoGen, USA) which contained whole length 270bp human SDF-1α cDNA driven by EF1-HTLV hybrid promoter with a downstream SV40 polyadenylation sequence (Figure S1). Preparation of the plasmid was performed per supplier’s instructions. Briefly, the disk of lyophilized GT100 E. coli bacteria transformed by pORF-hHSDF-1α was dissolved in E. coli FastMedia™ agar medium containing ampicillin. The expression vector had ampicillin resistance gene that allowed the selection of bacteria carrying the pORF plasmid. Plasmid DNA-hSDF-1α was purified with maxi-prep protocol (Qiagen, Valencia, CA, USA) per supplier's instructions. The purified plasmid was stored at − 20 °C until used for transfection of cells.

We used an expression vector containing hHGF gene (pBLAST49-hHGF) purchased from InvivoGen, USA contained a whole length 2187bp human HGF cDNA driven by EF1-HTLV hybrid promoter with a downstream SV40 polyadenylation sequence ( Figure S1). Preparation of the plasmid was performed per instructions of the supplier.

For construction of IGF and VEGF plasmids, we cloned 390bp and 576bp of their respective coding sequence amplified from human myoblast cDNA into pCMV-Script mammalian expression vector (Stratagene). Expression of the cloned genes was driven by a cytomegaloviral promoter sequence and downstream was an SV40 polyadenylation sequence (Figure S1). Preparation of the plasmids was performed using pCMV-Script PCR Cloning Kit (Stratagene, La Jolla, CA, USA Cat# 211199-12) per supplier’s instructions.

**Transfection of Sca-1^+^**

At 70–80% confluence, Sca-1^+^ were separately transfected with one of 4 plasmids (IGF-1, VEGF, SDF-1α, or HGF) using Polyethyleneimine (PEI, Polysciences Inc., Warrington, PA ,USA). Dissolve PEI power to a concentration of 2mg/ml in water which has been heated to 80℃. Allow solution to cool to room temperature and adjust pH to 7.0 with NaOH. To prepaire 50 μl transfection complexes: add DNA 3μg and PEI 10μl in DMEM mix by pipetting up-down. After Incubation for 8 minutes at room temperature, add 450ul complete medium(DMEM containing 10% FBS) to the mixture. Apply mixture to cells which have been washed previously with 2xPBS, mixed gently by rocking the plate back and forth every 15minutes and incubated for 2 hours at 37°C in a CO_2_ incubator. After removal of transfection complexes, transfected cells (^GF^Sca-1^+^)were cultured in DMEM containing 10% FBS 48 hours before pooling the different groups of cells transfected with their respective growth factor plasmid in one dish. The pooled ^GF^Sca-1^+^ were cultured at 37°C in a CO_2_ incubator for 24 hours before their use *in vitro* and *in vivo* studies. The transfected cells for each plasmid either alone or after pooling in on dish were characterized by fluorescence immunostaining, RT-PCR and western blotting.

**Reverse transcription polymerase chain reaction (RT-PCR)**

Isolation of total RNA from the different treatment cell groups, and their subsequent first strand cDNA synthesis was performed using RNeasy mini kit (Qiagen, Valencia, CA, USA) and Omniscript Reverse Transcription (Qiagen, Valencia, CA, USA) kit respectively per manufacturer’s instruction and described earlier ^[1]^. Amplification of the template DNA proceeded using the following thermocycle profile: initial denaturation for 3 minutes at 94°C; the 3-step cycling of denaturation for 1 minute at 94°C, annealing at respective time and temperatures and extension for 1 minute at 72°C, repeated for a total of 35 or 40 cycles; and a final extension for 10 minutes at 72°C. The primer sequences used for RT-PCR are given in Table SI. Densitometry was carried out using AlphaEase FC software (Alpha Innotech Corp).

**Fluorescent immunostaining of ^GF^Sca-1^+^**

^GF^Sca-1^+^ were fixed with 4% paraformaldehyde. The cells were incubated at 4°C overnight with anti-IGF1 (Santa Cruz Biotechnology Inc., CA USA), anti-VEGF (Santa Cruz Biotechnology Inc., CA USA), anti-SDF-1α (Cell Signaling Technology, MA, USA), or anti-HGF (R&D Systems, MN, USA) primary antibodies. After washing with PBS, they were incubated for 1 hour with respective secondary antibody conjugated with Alexa Fluor-546 (Molecular Probes, Invitrogen, Carlsbad, CA, USA). The immunostained cells were later observed using a fluorescent microscope (IX71 Olympus, Japan) fitted with a digital camera (Olympus, Japan).

**Western immunoblotting**

The cell samples from various groups were rinsed with PBS and then lysed with ice cold lysis buffer (50mM Tris-HCl, pH 7.6, 120mM NaCl, 0.5% NP-40, 1mM EDTA, 0.1mM. PMSF, 2μg/ml Leupeptin, 2μg/ml Aprotinin) on ice for 30 min. Cell lysates were centrifuged at 14000g for 10 minutes at 4ºC and the supernatants were collected. Protein concentration was determined by Protein Assay kit (BioRad, Berkeley, CA, USA). The protein samples were electrophoresed on a precast 4~12% gradient gel (Invitrogen, Calsbad, CA) using 20~50μg protein samples and electroimmunoblotted as described earlier[2]. The primary antibodies used for detection of the desired antigens have been listed in Table S2. Bands were visualized with super signal west femto and radiography film (Thermo Fisher Inc., Pierce, Rockford, Il USA).

**Cell migration and tube formation assays**

For preparation of the conditioned medium (CMD), 5x10^5^ ^GF^ Sca-1^+^ and ^SC^ Sca-1^+^ were separately cultured for 24 hours in 60 mm cell culture dishes in serum free basal DMEM supplemented with 0.05% BSA at 37^o^C. At the end of the stipulated time duration, the medium was removed and centrifuged at 250 G for 10 minutes to remove any cell debris. The supernatant thus obtained was used as a conditioned medium from ^GF^ Sca-1^+^ (^GF-Sca1^CMD) and ^SC^ Sca-1^+^ (^Sc-Sca1^CMD) in further experiments.

Conditioned medium were used for cell migration assay and tube formation assays. Briefly, for cell migration assay, human umbilical vein endothelial cells (HUVECs), suspended in a total volume of 0.5 ml serum-free DMEM containing 0.5% BSA, were seeded in the top well of a Transwell insert system (Corning Scientific Products, Acton, MA, USA) at a cell density of 50000 cells/well. CMD was added to the bottom wells of the transwell plates (8 μm pore size). Transwell chambers were incubated at 37°C and 5% CO_2_. After 6 hours incubation, transwell inserts were gently removed. The cells which failed to migrate remained on the upper side of the wells and were gently removed with cotton swab. The cells on the lower side of the transwell were fixed and stained with DAPI for 10 minutes. The membrane was dried and and emigrational activity was evaluated as the mean number of cells migrated to the bottom in 3-4 high power microscopic fields (200x) per chamber.

Tube formation assay was performed on Matrigel-coated 96-well plates to investigate whether CMD induced tube formation. HUVECs were seeded (1×10^4^ cells per well) on 96-well Matrigel plates with 200μl CMD collected from Sca-1^+^. The formation of tubular structures in the respective wells was examined at 16-hours after incubation of cells at 37°C in 5%CO_2_ with a phase-contrast microscope (IX71-Olympus, Japan).

**Cytoprotective activity of GF transfection**

To elucidate the cytoprotective effects of the GF transfection, the pooled ^GF^ Sca-1^+^ or ^GF^ Sca-1^+^ cocultured with neonatal cardiomyocytes (CM) were cultured under anoxia. Parallel experiments using ^SC^ Sca-1^+^ or ^SC^ Sca-1^+^ cocultured with neonatal CM were run as control. All experiments were performed in serum- and glucose-free DMEM. At the end of the experiment, cell supernatants were removed and used for lactate dehydrogenase (LDH) leakage as an indicator of cellular injury using LDH assay kit and the cells were used for TUNEL assay.

**LDH assay**

Intracellular LDH leakage was measured for each sample using Lactate Dehydrogenase Assay kit (CytoTox-One, Promega Corp., Madison USA;) per manufacturer’s instructions. Briefly, cell supernatant samples from different treatment groups were collected and centrifuged at 3000 rpm for 5 minutes to remove any cell debris. Set up 96-well assay plates containing cells supernatant 50μl/well. Add 50μl Substrate Mix Solution in all wells and mix briefly, then incubate for 10 minutes at room temperature (protect from light. Add 25μl stop solution to each well, shake plate for 10 seconds and record fluorescence with an excitation wavelength of 560nm and an emission wavelength of 590nm using Spectrophotometer (Beckman DU530, Life Science UV/Vis) .

**Histochemical and immunohistochemical studies**

For measurement of infarction size and area of fibrosis, the heart was arrested in diastole and fixed in 10% buffered formalin. The heart was then excised, cut transversely and embedded in paraffin. Histological sections of 5-μm thickness were cut and used for hematoxylin–eosin and Mason's trichrome staining for visualization of muscle architecture and area of fibrosis. Infarct size was defined as sum of the epicardial and endocardial infarct circumference divided by sum of the total LV epicardial and endocardial circumferences using computer based planimetry with Image-J analysis software (version 1.6065; NIH).

To determine the fate of the transplanted stem cells, histological tissue sections were immunostained with primary antibody to GFP (Abcam, Cambridge, MA, USA) and detected with secondary antibody conjugated to Alexa Fluor-488 (Molecular Probes, Invitrogen, Carlsbad, CA, USA). Blood vessel density was assessed by immunostaining for von Willebrand Factor-VIII (vWFactor-VIII) and detected with secondary antibodies conjugated to Alexa Fluor-546 (Molecular Probes, Invitrogen, Carlsbad, CA, USA). The number of blood vessels positive for vWFactor-VIII were counted in both infarct and peri-infarct regions. At least 5 microscopic fields in infarct and peri-infarct regions each animal were randomly selected and counted in each treatment group. Blood vessel density was expressed as the number of vessels per microscopic surface area at 200X magnification. The total number of stem/progenitor cells that homed into the infarct and peri-infarct areas of the heart was determined by counting the cells positive for the respective surface marker at least 5 microscopic field per slide from each heart. The primary antibodies used for detection of the desired antigens have been listed in Table S2.

**The heart function studies**

The left ventricle heart function and dimension measurements were carried out at 4 weeks after cell transplantation as described earlier [3]. The animals (n = 7 per group) were anesthetized and lightly secured in the supine position on a warm pad. After the chest was shaven, Acoustic gel was applied and transthoracic echocardiography was performed using HDI-5000 SONOS-CT (HP) ultrasound machine with a 7-MHz transducer. The heart was imaged in the two-dimensional mode in the parasternal long-axis and/or parasternal short-axis views which were subsequently used to position the M-mode cursor perpendicular to the ventricular septum and left ventricle posterior wall, after which M-mode images were obtained. For each animal, measurements were obtained from 3 consecutive heart cycles. Measurements of ventricular septal thickness (VST), left ventricle internal dimension (LVID), and left ventricle posterior wall thickness (LVPW) were made from two-dimensionally directed M-mode images of the left ventricle in both systole and diastole. The average value from all measurements in an animal were used to determine the indices of left ventricle contractile function, i.e., left ventricle fractional shortening (LVFS) and left ventricle ejection fraction (LVEF) using the following relations LVFS = (LVEDd−LVESd)/LVEDd×100 and LVEF = [(LVEDd^3^−LVESd^3^)/LVEDd^3^]×100 and expressed as percentages.

## Isolation of neonatal cardiomyocytes

Neonatal cardiomyocytes were isolated from newborn (1-3 day) Fisher-344 rat pups using Worthington Neonatal Cardiomyocytes Isolation System (Worthington Biochemical, NJ, USA) following to the manufacturer’ protocol. Briefly, pups were kept on ice for 5-10 minutes. After soaking in 70% ethanol, an incision was made to the left side of the sternum and the heart was removed with the help of forceps. Upon harvesting, the hearts were minced to small pieces and enzymatically digested by incubation overnight at 4^o^C in trypsin 50µg/ml. The tissue was then oxygenated for 1 minute and incubated with 5 ml collagenase at concentration of 300units/ml on the shaker (2-4 rounds per minute) for 45 minutes at 37^o^C. Following this, the tissue was oxygenated and triturated several times, and filtered through 70µm strainer to remove the undigested tissue. The cells were allowed to settle for 20 minutes at room temperature and centrifuged at 100G for 5 minutes before pre-plating twice (45 minutes each) in DMEM media (Hyclone, Logan, UT, USA) supplemented with 10% FBS (Hyclone, Logan, UT, USA) and Penicillin /Streptomycin at 37^o^C. At the end of pre-plating, the cardiomyocyte enriched cell preparation was counted for cell number using haemocytometer and cells were later plated for use in various experiments.

**Formation of gap-junction between ^GF^ Sca-1^+^**  **and CM**

^GF^ Sca-1^+^  or ^SC^ Sca-1^+^  (GFP negative cells labled with DAPI) were co-cultured with neonatal CM at 1:10 ratio in 35mm dish for 48 hours. The cells were later fixed with 4% paraformaldehyde and immunostained for Cx43 using anti-CX43 (rabbit anti-rat, 1: 50 dilution, Santa Cruz, CA, USA). The primary antigen-antibody reaction was detected with Alexa 546 conjugated anti-rabbit antibody (1:200 dilution; Invitrogen, Carlsbad, CA, USA). The nuclei were visualized with SYTOX Green 0.25µM (Invitrogen, Carlsbad, CA, USA). The dishs were mounted with Fluoromount-G (SouthernBiotech, Birmingham, AL. USA) and observed under fluorescent microscope (IX71 Olympus, Japan) for 5 fields per dish at 400x magnification.

**References**

1. Lu G, Haider HK, Jiang S, Ashraf M (2009) Sca-1+ stem cell survival and engraftment in the infarcted heart: dual role for preconditioning-induced connexin-43. Circulation 119:2587-2596.
2. Niagara MI, Haider H, Jiang S, Ashraf M (2007) Pharmacologically preconditioned skeletal myoblasts are resistant to oxidative stress and promote angiomyogenesis via release of paracrine factors in the infarcted heart. Circ Res 100:545-555.
3. Pasha Z, Haider K H, M Ashraf (2011) Efficient non viral reprogramming of myoblasts to stemness with a single small molecule for generating cardiac progenitors cells. PLoS One 6:e23667
